# Mixtures of Three Mortaparibs with Enhanced Anticancer, Anti-Migration, and Antistress Activities: Molecular Characterization in p53-Null Cancer Cells

**DOI:** 10.3390/cancers16122239

**Published:** 2024-06-17

**Authors:** Renu Wadhwa, Shi Yang, Hazna Noor Meidinna, Anissa Nofita Sari, Priyanshu Bhargava, Sunil C. Kaul

**Affiliations:** AIST-INDIA DAILAB, National Institute of Advanced Industrial Science & Technology (AIST), Central 4-1, Tsukuba 305-8565, Japan

**Keywords:** Mortaparib, Mortaparib^Plus^, Mortaparib^Mild^, downregulation, mortalin, PARP1, p53-null cells, anti-proliferation, anti-migration, antistress, therapy

## Abstract

**Simple Summary:**

The Hsp70 family stress chaperone mortalin plays an important role in carcinogenesis and hence has emerged as a candidate target for cancer drug development. Mortaparibs (Mortaparib, Mortaparib^Plus^, and Mortaparib^Mild^) are chemically synthesized small molecules isolated as inhibitors of mortalin–p53 interaction. They also downregulate mortalin and PARP1 expression and block cancer signaling in multiple ways. In this study, we demonstrate (i) the anticancer activity of Mortaparib^Mild^ in p53-null cancer cells and (ii) combinations of three Mortaparibs for enhancing their potency for treating the hyper-proliferation, -migration, and -stress phenotypes of cancer cells.

**Abstract:**

Mortalin, a member of the Hsp70 family of proteins, is commonly enriched in many types of cancers. It promotes carcinogenesis and metastasis in multiple ways of which the inactivation of the tumor suppressor activity of p53 has been firmly established. The downregulation of mortalin and/or disruption of mortalin–p53 interactions by small molecules has earlier been shown to activate p53 function yielding growth arrest/apoptosis in cancer cells. Mortaparibs (Mortaparib, Mortaparib^Plus^, and Mortaparib^Mild^) are chemical inhibitors of mortalin isolated by cell-based two-way screening involving (i) a shift in the mortalin staining pattern from perinuclear (characteristics of cancer cells) to pancytoplasmic (characteristics of normal cells) and (ii) the nuclear enrichment of p53. They have similar structures and also cause the inhibition of PARP1 and hence were named Mortaparibs. In the present study, we report the anticancer and anti-metastasis activity of Mortaparib^Mild^ (4-[(4-amino-5-thiophen-2-yl-1,2,4-triazol-3-yl)sulfanylmethyl]-N-(4-methoxyphenyl)-1,3-thiazol-2-amine) in p53-null cells. By extensive molecular analyses of cell proliferation, growth arrest, and apoptosis pathways, we demonstrate that although it causes relatively weaker cytotoxicity compared to Mortaparib and Mortaparib^Plus^, its lower concentrations were equally potent to inhibit cell migration. We developed combinations (called Mortaparib^Mix-AP^, Mortaparib^Mix-AM^, and Mortaparib^Mix-AS^) consisting of different ratios of three Mortaparibs for specifically enhancing their anti-proliferation, anti-migration, and antistress activities, respectively. Based on the molecular analyses of control and treated cells, we suggest that the three Mortaparibs and their mixtures may be considered for further laboratory and clinical studies validating their use for the treatment of cancer as well as prevention of its relapse and metastasis.

## 1. Introduction

Cancer is a highly heterogeneous fatal disease showing exponential increase worldwide. Based on its diverse etiology, potential to spread throughout the body, and resistance to drugs, it warrants multifaceted diagnostic and treatment modalities. The dysregulation of tumor suppressor proteins involved in the control of cell proliferation, migration, invasion, and drug response has been established as a key event in cancer origin and progression to aggressive stages. These largely include p53, p21, pRB, p16, p19, PTEN, Keap1, NRF2, PARP1, Her2, and CDKs that have emerged as therapeutic targets in various types of cancers [1,2,3,4]. Driven by the functional inactivation of a large number of tumor suppressor genes and activation of oncogenes, it involves a complex network of multiple signaling cascades including replication and cell cycle progression, stress and DNA damage response, cell migration, and EMT that are also modulated by intrinsic and extrinsic stresses [5,6]. Oxidative stress, often defined by the accumulation of reactive oxygen species (ROS), a normal by-product of cell survival pathways, plays a critical role in carcinogenesis and its progression to lethal stages. The aberrant accumulation of ROS affects genomic stability and the microenvironment and disrupts cellular homeostasis leading to the loss of normal cellular functions. Molecular chaperones that regulate stress response as a first line of defense by attenuating ROS and facilitating the correct conformation and intracellular localization of proteins have been considered therapeutic targets for age-related metabolic diseases and cancer [7,8]. The latter is commonly associated with the overexpression of stress chaperones (including HSP40, HSP60, HSP70, and HSP90) that play a substantial role in hyper-proliferation and anti-apoptotic characteristics of tumors and hence are also used as tumor biomarkers [9]. Besides their overexpression, the subcellular localization of these molecular chaperones is modulated during physiologically stressful conditions and cancer. For example, Hsp70 is localized in the cell membrane or released in extracellular vesicles in malignant tumors [10]. The Hsp70 family member mortalin is enriched in many types of cancers and plays a key role in regulating the proliferation, migration, and invasive properties of cancer cells [11,12,13,14,15,16,17]. It exhibits pancytoplasmic subcellular distribution in normal and perinuclear niches in cancer cells [11]. The induction of senescence by ribozymes, siRNA, or phytochemicals was marked by a shift in its subcellular localization from perinuclear to pancytoplasmic in cancer cells that showed growth arrest, apoptosis, and the reactivation of the transcriptional activation function of the p53 tumor suppressor [11,18,19,20]. Furthermore, mortalin localizes in the nucleus of cancer cells wherein it activates telomerase and heterogeneous ribonucleoprotein K (hnRNP-k) proteins [11] involved in malignant transformation. It stimulates epithelial–mesenchymal transition (EMT) and cancer cell stemness by upregulating the expression levels of mesenchymal markers including vimentin, fibronectin, β-catenin, CK14, and hnRNP-k [11,14,19,20,21,22]. Furthermore, it was also shown to promote oncogenic characteristics of KRAS and BRAF tumor cells specifically by modulating the MEK/ERK pathway and serving as a selective therapeutic target for KRAS-mutated tumors [23,24].

In the above premise, mortalin expression and its interactions with p53 have emerged as a cancer drug target [11,14,19,20,21]. We previously screened a small molecule library consisting of 12,000 compounds by a cell-based multiple assay platform including (i) a shift in mortalin distribution from perinuclear to pancytoplasmic and (ii) the nuclear translocation and reactivation of wild-type p53 function. Three top hits of this screening were the novel molecules that we named Mortaparib [25], Mortaparib^Plus^ [26,27], and Mortaparib^Mild^ [28] due to their functions including the inhibition of mortalin, mortalin-p53 interaction, and PARP1. Subsequent studies have supported the idea that the action of these molecules is not restricted to the wild-type p53-harboring cancer cells. Mortaparib^Plus^ was shown to be more potent and target PARP1, p73, and CARF proteins, critically involved in carcinogenesis and metastasis [26,27]. Mortaparib^Mild^ inhibited mortalin and PARP1 functions, although it showed a low level of cytotoxicity [28]. In the present study, we examined the response of p53-null cancer cells to Mortaparib^Mild^. Both cell types responded to Mortaparib^Mild^ through the downregulation of mortalin and PARP1 expression. It caused a significant inhibition of cell migration and invasion characteristics, comparable to the other two Mortaparibs. Based on these results, we developed a combination of three Mortaparibs (Mortaparib^Mix^) for enhancing anti-proliferation (Mortaparib^Mix-AP^) potency for cancer therapy. Furthermore, anti-migration (Mortaparib^Mix-AM^) and antistress (Mortaparib^Mix-AS^) combinations were generated and validated for their consideration in treating cancer metastasis and its stress-related progression or relapse.

## 2. Materials and Methods

### 2.1. Cells and Reagents

Human osteoblastoma (U2OS and Saos2) and ovarian adenocarcinoma (SKOV3) were obtained from Japanese Collection of Research Bioresources (Tokyo, Japan) and cultured in Dulbecco’s Modified Eagle’s Medium (DMEM: low glucose with L-glutamine and phenol red) supplemented with 5–10% fetal bovine serum (Fujifilm WAKO Pure Chemical Corporation, Osaka, Japan) and 1% penicillin–streptomycin at 37 °C in a humidified incubator with 5% CO_2_. Drug-resistant Saos2 cells were obtained by culturing them in DMEM supplemented with 0.5 µM cisplatin for three months with a 1:16 splitting ratio and regular change of medium every third day. The growth and morphology of cells were monitored every day. Cells were examined for their response to cisplatin by MTT assay (every 2–3 weeks) as described below. Cells that showed 10–20% resistance to cisplatin were stored in a −80 °C freezer and used for further experiments.

Mortaparib (5-[1-(4-methoxyphenyl)(1,2,3,4-tetraazol-5-yl)]-4-phenylpyrimidine-2-ylamine) [25], Mortaparib^Plus^ (4-[(1E)-2-(2-phenylindol-3-yl)-1-azavinyl]-1,2,4-triazole) [26,27], and Mortaparib^Mild^ (4-[(4-amino-5-thiophen-2-yl-1,2,4-triazol-3-yl)sulfanylmethyl]-N-(4-methoxyphenyl)-1,3-thiazol-2-amine) [28] were purchased from NAMIKI SHOJI Co., Ltd. (Shinjuku, Japan). Stock solutions (50 mM) were prepared in Dimethyl Sulfoxide (DMSO) (WAKO, Osaka, Japan) and were diluted in complete cell culture media to obtain concentrations from 5 to 30 µM. Cells were treated at 60–70% confluency for 24–48 h.

### 2.2. Cell Viability Assay

Cytotoxicity was determined by MTT (3-(4,5-dimethylthiazol-2-yl)-2,5-diphenyltetrazolium bromide) assay (Sigma Aldrich, Tokyo, Japan). Cells (5 × 10^3^/well) were seeded in a 96-well plate, allowed to settle overnight, and then treated with each of the three Mortaparibs (Mortaparib, Mortaparib^Plus^, Mortaparib^Mild^) or their combination for 48 h at concentrations as shown in Figures. In each case, cells were treated with DMSO as a solvent control. The MTT solution (100 μL, 5 mg/mL) was added to each well, with continued incubation at 37 °C for 3–4 h, followed by the removal of the MTT solution and the addition of 100 µL of DMSO for terminating the reaction. The plates were left on slow shaking for 20 min at room temperature for the complete solubilization of the MTT-formazan product. The absorbance of the latter was measured at 570 nm using an Infinite M200 Pro microplate reader (Tecan Group Ltd., Mannedorf, Switzerland) and converted to the percent cell viability of the treated vs. control groups.

### 2.3. Colony Formation Assay

The long-term (2 weeks) cytotoxicity of Mortaparibs was determined by colony formation assay. Saos2 and SKOV3 cells (5 × 10^2^/well) were plated in 6-well plates and allowed to settle overnight followed by treatment with complete media containing DMSO (control) or Mortaparib^Mild^ (1–20 µM). A regular change of medium was performed every third day until colonies appeared and showed a gradual increase in size until they covered about 80% of the surface of the control culture plate. Colonies were washed with cold PBS, fixed with methanol–acetone (1:1, *v*/*v*), and stained with 0.1% crystal violet solution (Wako, Osaka, Japan) at room temperature, followed by destaining with MilliQ and air-drying. The colony formation capacity was determined by counting the colonies.

### 2.4. Cell-Cycle Analysis

Saos2 and SKOV3 cells were seeded in the 6-well plates (18 × 10^4^ cells/well). After 24 h, control and Mortaparib^Mild^-treated cells were harvested, centrifuged at 2000 rpm at 4 °C for 5 min, washed with cold PBS, fixed with 70% ethanol on slow vortex, and kept at −20 °C for up to 72 h. The fixed cells were centrifuged at 3000 rpm at 4 °C for 10 min followed by two more cycles of cold PBS washing. The cells were stained with Guava Cell Cycle Reagent (4500-0220) (Luminex Corporation, Austin, TX, USA) in the dark for 30 min. The samples were treated with RNase-A (1 mg/mL at 37 °C for 30 min) to eliminate RNA. Cell cycle progression was examined using the Guava PCA-96 System (Luminex Corporation, Austin, TX USA). FlowJo software (Version 7.6, Flow Jo, LLC, Ashland, OR, USA) was used to analyze the flow cytometry data.

### 2.5. Western Blot Analysis

Cells (2 × 10^5^/well) were plated in 6-well plates and allowed to settle overnight followed by culture in DMEM supplemented with either solvent (DMSO) or Mortaparibs (48 h) followed by harvest by trypsinization. Cell pellets were lysed in RIPA Lysis Buffer (Thermo Fisher Scientific, Waltham, MA, USA) supplemented with a complete protease inhibitor cocktail (Roche Applied Science, Mannheim, Germany) for 30 min at 4 °C with gentle vortex. The lysates were centrifuged at 15,000 rpm for 15 min, and the supernatant was used to estimate protein concentration by BCA (Bicinchoninic acid) assay (Thermo Fisher Scientific, Waltham, MA, USA). Supernatants containing 10–20 µg protein were separated using 8–12% SDS–polyacrylamide gel electrophoresis (SDS-PAGE) and transferred onto a polyvinylidene difluoride (PVDF) membrane (Millipore, Billerica, MA, USA) using either a wet transfer system (Mini-PROTEAN Tetra Cell, BIO-RAD, Hercules, CA, USA) for ~60 min or a semi-dry transfer blotter (ATTO Corporation, Tokyo, Japan) for ~45 min. The PVDF membranes were blocked using a 3% bovine serum albumin at room temperature for 1 h. The membranes were then incubated overnight at 4 °C with primary antibodies specific to the target proteins. Details of all primary antibodies are provided in Supplementary Table S1. Subsequently, the blots were washed with TBST (0.2% Tween 20 in TBS) 3 times (10 min each at room temperature) and probed with matched secondary antibodies (anti-rabbit IgG, 31460 or anti-mouse IgG, 31430; Thermo Fisher Scientific) followed by 3–5 washings with TBST. Direct-blot anti-β-actin antibody (BioLegend) was used as an internal loading control. Blots were developed using enhanced chemiluminescence reaction (ECL) (GE Healthcare, Buckinghamshire, UK). The protein band images were obtained using Gel Doc Documentation (Bio-Rad, CA, USA). The band intensity was quantified using ImageJ 1.53k software (National Institutes of Health, Bethesda, MD, USA) and plotted as relative units of expression.

### 2.6. Immunocytochemistry

Cells (1.5 × 10^5^/well) were plated on 18 mm glass coverslips placed in 12-well plates, allowed to settle overnight, and treated with DMEM or Mortaparibs, as indicated, for 48 h followed by fixation in pre-chilled methanol–acetone (1:1) on ice for 10 min. Fixed cells were permeabilized with PBST (PBS with 0.2% Triton-X) for 10 min, washed with PBS for 10 min, blocked with bovine serum albumin (2% in PBST) for 60 min, and then incubated with primary antibodies overnight. Coverslips were extensively washed with PBST (thrice, 10 min each) and then incubated with secondary antibodies for 2 h. Alexa-Fluor 488 goat anti-mouse IgG (Life Technologies, A11029, Carlsbad, CA, USA) and Alexa-Fluor 594 goat anti-rabbit IgG (Life Technologies, A11037) were used as the secondary antibodies. After washing with PBST (thrice, 10 min each), coverslips were incubated with Hoechst 33342 (Invitrogen, H3570, Waltham, MA, USA) for the nuclear staining of cells, washed with PBST, mounted with Fluoromount Aqueous Mounting Medium (Sigma-Aldrich, F4680, St. Louis, MO, USA). Immunofluorescence was examined under a microscope (Zeiss Axiovert 200 M) and analyzed by AxioVision 4.6 software (Carl Zeiss, Oberkochen, Germany). Protein expression was quantified using ImageJ software (NIH, Bethesda, MD, USA) and plotted as relative units of expression against the control group.

### 2.7. RNA Extraction and Real-Time Quantitative Polymerase Chain Reaction (RTqPCR)

Total RNA from control and Mortaparib^Mild^-treated cells was collected by RNeasy mini kit (Qiagen, Stanford Valencia, CA, USA) following the manufacturer’s protocol. Equal amounts of RNA (1 µg) from samples were reverse-transcribed into cDNA following the protocol from the QuantiTect Reverse Transcription kit (Qiagen, Tokyo, Japan). Real-time quantitative Polymerase Chain Reaction (RTqPCR) was performed using the protocols for SYBR Select Master Mix (Applied Biosystem, Life Technologies, Foster City, CA, USA) using mortalin primers (Forward—AGCTGGAATGGCCTTAGTCAT and Reverse—CAGGAGTTGGTAGTACCCAAATC). The conditions of RT-qPCR were 50 °C for 2 min, 95 °C for 10 min, followed by 40 cycles (denaturation at 95 °C for 15 s, annealing at 60 °C for 1 min, and extension at 72 °C for 15 s). The relative expression level of the target genes was normalized against the 18S gene (Forward primer—CAGGGTTCGATTCCGTAGAG and Reverse primer—CCTCCAGTGGATCCTCGTTA) as an internal control.

### 2.8. Trapping Assay

Control and Mortaparib^Mild^-treated cells were collected by centrifugation (2500 rpm at 4 °C for 3 min). Supernatants were removed, and pellets were mixed with hypotonic buffer (constituted for different stringency as shown in Table S2) and then vortexed for 10 min followed by centrifugation at 16,000 rpm at 4 °C for 10 min. The collected supernatant was labeled as P1, and the pellet was re-suspended with Buffer A. This step was serially repeated in the sequence of buffer A–D. Supernatants from each centrifugation step were collected and labeled as A, B, C, and D. Western blotting was performed using anti-PARP1/2 and anti-histone H3 antibodies.

### 2.9. Wound Healing Assay

Cells (1.5 × 10^5^/well) were plated in a 6-well plate and allowed to settle overnight in a CO_2_ incubator at 37 °C. To create a wound, a sterile pipette tip (200 μL) was used to make a linear scratch. Cells were washed with PBS three times to remove any floating cells and were then subjected to either DMEM or the medium supplied with Mortaparibs (as indicated). The migration of cancer cells into the scratch was monitored throughout 0 to 48 h, and the images at different time-points were captured under the microscope (Nikon TS100-F, Tokyo, Japan). The width of the scratch was determined using ImageJ software (NIH, Bethesda, MD, USA).

### 2.10. Invasion Assay

BioCoatTM Matrigel Invasion kit (354480; Corning, Labware, Inc., Two Oak Park, MA, USA) was used. Cells (5 × 10^4^/well) were suspended in 0.5 mL of serum-free DMEM and introduced into the upper chamber of invasion inserts placed in a 24-well culture plate. Concurrently, the lower chamber of the plate was filled with 0.75 mL of DMEM supplemented with 10% FBS. Following 48 h incubation, the chambers were transferred to fresh plates and washed thrice with PBS. Cells that had invaded, becoming entrapped in the Matrigel basement membrane matrix at the base of each insert, were fixed using methanol–acetone solution (1:1, *v*/*v*) and stained overnight with 0.5% Crystal Violet. The excess stain was removed by rinsing with ultrapure water. The chambers were air-dried and photographed under a microscope (Nikon TS100-F, Tokyo, Japan). The percentage of invaded cells was quantitated using ImageJ software (NIH, Bethesda, MD, USA).

### 2.11. Reactive Oxygen Species (ROS) Assay

The Image-ITTM LIVE Green Reactive Oxygen Species Detection Kit (Molecular Probes, Eugene, OR, USA) was used for the ROS assay, adhering to the guidelines provided by the manufacturer. Cells (5 × 10^4^/well) were seeded on glass coverslips (18 mm), placed in 12-well plates, and allowed to settle overnight. After a 24 h exposure to hydrogen peroxide, the cells were cultured in either control or Mortaparib-supplemented DMEM for 48 h. Images were captured under a Carl Zeiss microscope (Axiovert 200M) and subjected to analysis through AxioVision 4.6 software (Carl Zeiss). The quantification of ROS fluorescence intensity was performed using Image J software (NIH, Bethesda, MD, USA).

### 2.12. Mitochondrial Membrane Potential [ΔΨm] Assay

The detection of mitochondrial membrane potential in control and treated cells was performed by JC-1 staining dye (ab141387, Abcam, Boston, MA, USA). Cells (6 × 10^4^/well) were seeded on 18 mm glass coverslips placed in 12-well plates treated with hydrogen peroxide for 24 h followed by recovery either in control or Mortaparib-supplemented medium for 48 h. Cells were then subjected to JC-1 dye (10 μg/mL) for 30 min in a CO_2_ incubator at 37℃, followed by washing with PBS. The fluorescence of JC-1 monomers (green) and aggregates (red) was observed using a Carl Zeiss microscope (Axiovert 200M), and the fluorescence intensity ratio of aggregates to monomers was analyzed through Image J software (NIH, Bethesda, MD, USA).

### 2.13. Statistical Analysis

Data from three or more independent experiments were statistically expressed as the mean ± standard deviation. An unpaired *t*-test (GraphPad Prism 10.2.3.403, San Diego, CA, USA) was performed to determine the statistical significance between the two groups (control and treated). In experiments involving three groups such as control, stressed, and recovered, the data were quantitated against the stressed group. Significance levels were categorized as follows: *p* > 0.05 (ns) for non-significant, *p* ≤ 0.05 (*) for statistical significance, *p* ≤ 0.01 (**) for very significant, *p* ≤ 0.001 (***) for highly significant, and *p* ≤ 0.0001 (****) for extremely significant, respectively. The synergetic effect of the combination was determined by Chou–Talalay’s combination index (CI) using CompuSyn 1.0 (ComboSyn Inc., Paramus, NJ, USA). The CI score represents the quantitative determination of the synergism (CI < 1), antagonism (CI > 1), and additive effect (CI = 1) of the drug combination.

## 3. Results

### 3.1. Mortaparib^Mild^ Caused Growth Arrest of p53-Null Cancer Cells

**Figure 1:**
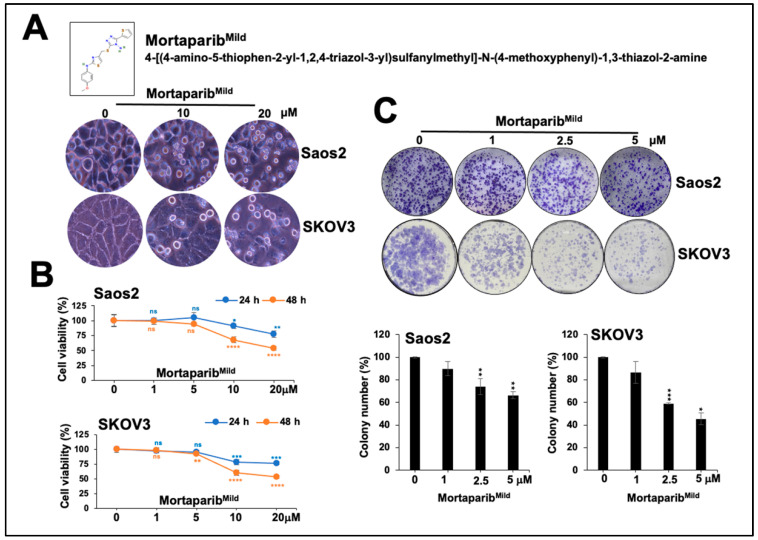
Cytotoxicity of Mortaparib^Mild^ in p53-null cells. (**A**) Chemical structure of Mortaparib^Mild^ is shown. Cells treated with Mortaparib^Mild^ for 48–72 h showed apoptotic morphology. (**B**) MTT assay showed dose-dependent cytotoxicity in both Saos2 and SKOV3 cells. (**C**) Clonogenicity of control and treated cells as determined by colony forming assay showing dose-dependent decrease in Mortaparib^Mild^-treated group. Quantification of data from three replicates with *p*-values calculated by unpaired Student’s *t*-test is shown; *p* ≥ 0.05 (ns), *p* ≤ 0.05 (*), *p* ≤ 0.01 (**), *p* ≤ 0.001 (***), *p* ≤ 0.0001 (****) represent non-significant, significant, very significant, highly significant, and extremely significant, respectively.

We examined the effect of Mortaparib^Mild^ on Saos2 and SKOV3 cells (p53 null, as also endorsed by immunostaining; Supplementary Figure S1A). In parallel assays, no change was observed in either Saos2 or SKOV3 cells, whereas U2OS cells (p53 wild-type) treated with Mortaparib^Mild^ showed an increase in nuclear p53, as also reported earlier [28]. However, as shown in Figure 1, both Saos2 and SKOV3 cells treated with 10–20 μM Mortaparib^Mild^ for 24–48 h showed apoptotic morphology (Figure 1A) and a dose-dependent decrease (25 to 50%) in the cell viability assay (Figure 1B). We performed cell cycle analysis and detected a dose-dependent increase in cells at G1 and G2 stages in Saos2 and SKOV3 cultures treated with 5 and 10 μM Mortaparib^Mild^, respectively (Supplementary Figure S1B). Annexin assay revealed a progressive increase in early apoptotic cells in Mortaparib^Mild^-treated Saos2 and SKOV3 cultures (Supplementary Figure S1C). The long-term (2 weeks) effect, as examined by colony forming assay, showed a decrease in clonogenicity in Mortaparib^Mild^-treated cells (~15, 30, and 40% in Saos2 and ~20, 40, and 55% in SKOV3 with 1, 2.5, and 5 μM Mortaparib^Mild^, respectively (Figure 1C)). These data suggested that Mortaparib^Mild^ is effective for p53-null cancer cells in addition to its p53-dependent cytotoxicity in p53 wild-type cancer cells [28]. We investigated the mechanism of its cytotoxicity in p53-null cells as follows.

**Figure 2:**
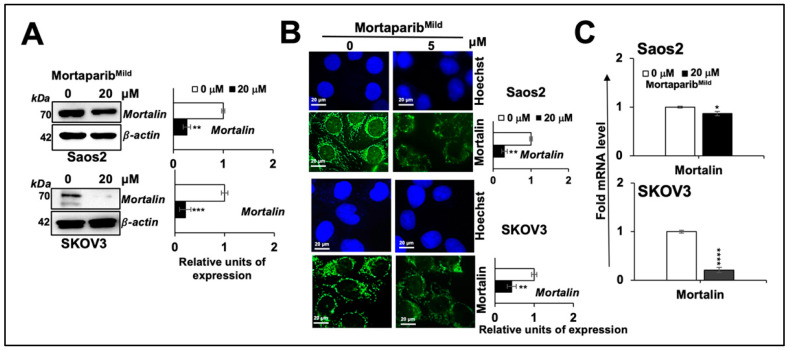
Mortaparib^Mild^ induced downregulation of mortalin in p53-null cells. (**A**) Western blotting and (**B**) immunostaining of mortalin in control and treated cells showed decrease in latter. Quantitation is shown on right. Quantification of data from three replicates with *p*-values calculated by unpaired Student’s *t*-test is shown; *p* ≤ 0.05 (*), *p* ≤ 0.01 (**), *p* ≤ 0.001 (***), *p* ≤ 0.0001 (****) represent significant, very significant, highly significant, and extremely significant, respectively. The uncropped blots are shown in File S1. (**C**) Mortalin mRNA expression in control and treated cells, as determined by RT-qPCR, is shown.

**Figure 3:**
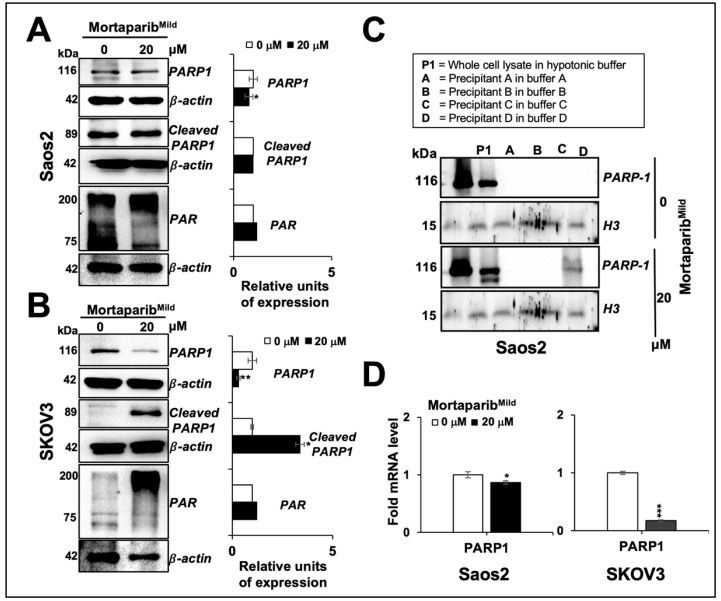
Mortaparib^Mild^ induced inactivation of PARP1 in p53-null cells. (**A**,**B**) Mortaparib^Mild^-treated cells showed reduction in full-length PARP1 in both cell types. Cleaved 89 kDa fragment of PARP1 and PAR polymer showed remarkable increase in SKOV3 (**B**). DNA trapping assay showed inactivation of PARP1 (**C**). β-actin (**A**,**B**) and histone H3 (**C**) were used as internal loading controls in Western blotting. (**D**) PARP1 mRNA expression in control and treated cells as determined by RTqPCR is shown. Quantified data represent mean ± SD obtained from independent biological replicates; *p*-values were calculated using unpaired Student’s *t*-test. * ≤ 0.05, ** ≤ 0.01, and *** ≤ 0.001 represent significant, very significant, and highly significant, respectively. The uncropped blots are shown in File S1.

Mortaparib^Mild^-treated p53-null cancer cells were first examined for the expression level of mortalin. As shown in Figure 2A*,*B, Western blotting and immunostaining on control and Mortaparib^Mild^-treated Saos2 and SKOV3 cells showed a decrease in mortalin upon treatment with Mortaparib^Mild^. Furthermore, mortalin mRNA showed a decrease that was more pronounced in SKOV3 than Saos2 cells (Figure 2C). Similar to mortalin, PARP1 expression was reduced in Mortaparib^Mild^-treated Saos2 cells (Figure 3A). It was comparable to the p53 wild-type cells reported earlier [28] and particularly showed a remarkable decrease in SKOV3 cells with a significant increase in cleaved PARP1 (Figure 3B). DNA trapping assay endorsed the attenuation of PARP1 activity in Mortaparib^Mild^-treated Saos2 (Figure 3C). PARP1-RTqPCR showed a higher reduction in PARP1 mRNA in SKOV3 cells (Figure 3D) that was consistent with the results on protein expression.

### 3.2. Mortaparib^Mild^ Caused Inhibition of Metastatic Properties of p53-Null Cancer Cells

**Figure 4:**
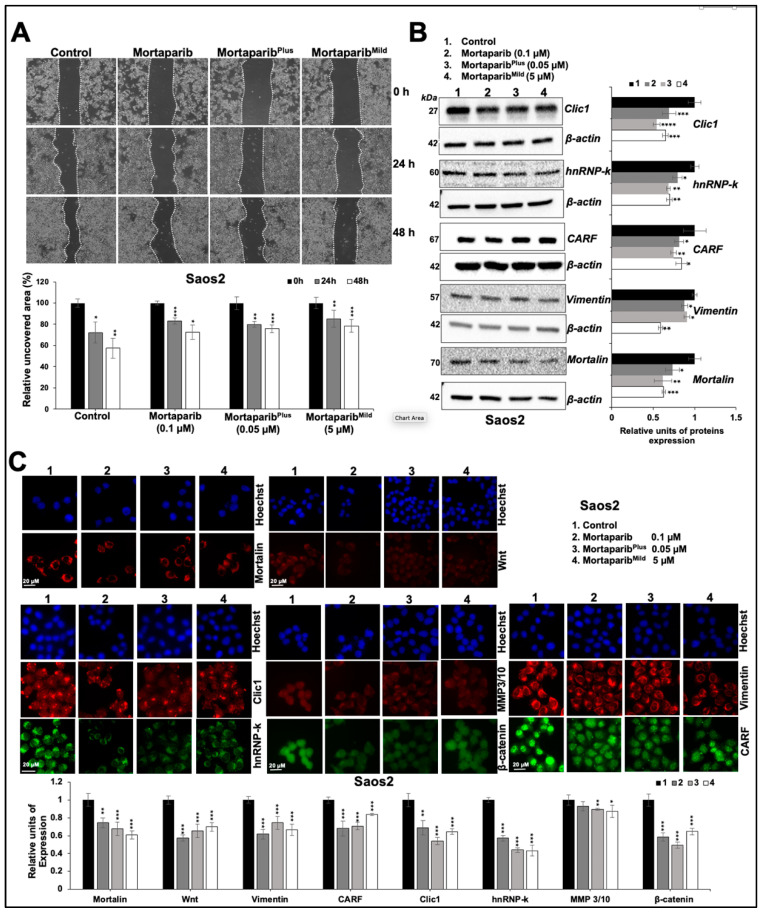
Three Mortaparibs showed comparable anti-migration activity. (**A**) Wound scratch assay in Saos2 cells treated with non-toxic doses of Mortaparibs is shown. All three treated groups showed delay in migration, as compared to control at both 24 h and 48 h time-points. Quantitation of data is shown below. (**B**) Molecular markers of migration in Western blotting of control and treated cells showed comparable decrease in treated as compared to control. Quantification is shown on right. The uncropped blots are shown in File S1. (**C**) Molecular markers of migration in immunostaining of control and treated cells showed a decrease in treated as compared to control. Quantification is shown below. Data, shown in graphs (mean ± SD), were obtained from three independent biological replicates. *p*-values were calculated using unpaired Student’s *t*-test. * ≤ 0.05, ** ≤ 0.01, *** ≤ 0.001, **** ≤ 0.0001 represent significant, very significant, highly significant, and extremely significant, respectively.

**Figure 5:**
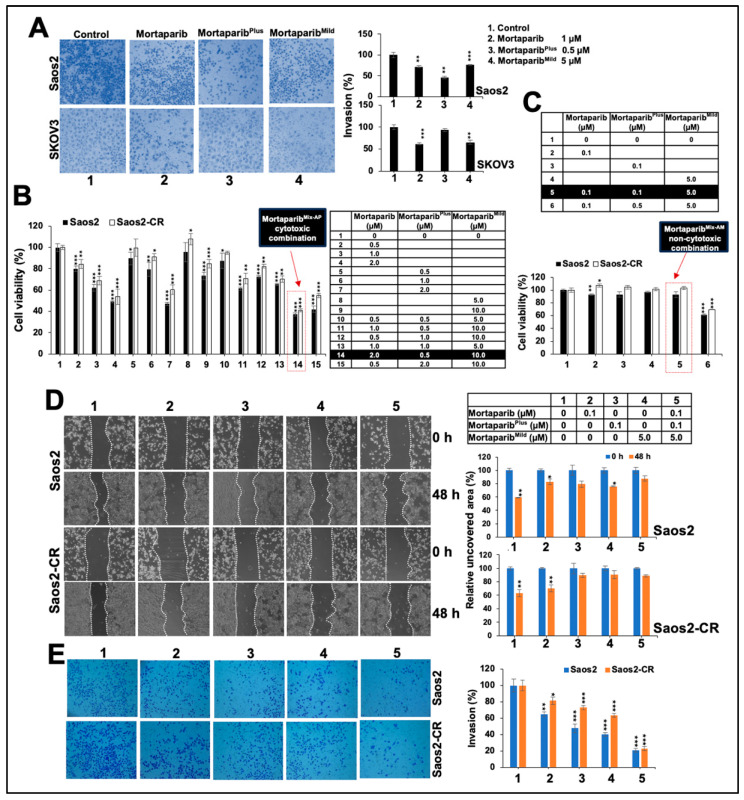
Three Mortaparibs showed comparable anti-invasion activity. (**A**) Invasion assay with non-toxic doses of three Mortaparibs in Saos2 and SKOV3 showed inhibition in treated compared to control groups. Quantitation of data is shown on right. (**B**) Viability of Saos2 and its drug-resistant derivative cell line treated with each of three Mortaparibs (#1–9) and their mixtures (#10–15); #14 showed higher cytotoxicity to both cell types. (**C**) Viability of cells treated with low doses of Mortaparibs (#1–4) and their mixture (#5–6); #5 was selected as non-toxic mixture. *p*-values were calculated using unpaired Student’s *t*-test. * ≤ 0.05, ** ≤ 0.01, and *** ≤ 0.001 represent significant, very significant, and highly significant, respectively. (**D**) Wound scratch assay in Saos2 and Saos2-CR cells treated with non-toxic doses of three Mortaparibs and their mixture. (**E**) Cell invasion assay in Saos2 and Saos2-CR cells treated with non-toxic doses of three Mortaparibs and their mixture. Mixture #5 showed higher anti-migration and anti-invasion activity. Data (mean ± SD) were obtained from three independent biological replicates. *p*-values were calculated using unpaired Student’s *t*-test. * ≤ 0.05, ** ≤ 0.01, and *** ≤ 0.001 represent significant, very significant, and highly significant, respectively.

We next examined the effect of Mortaparib^Mild^ on the metastatic properties of Saos2 and SKOV3 cells. In both cell types, the low concentrations (1–5 μM) of Mortaparib^Mild^ that did not affect cell proliferation (Supplementary Figure S2A) caused a delay in migration at both 24 h and 48 h time-points as detected by wound scratch assays (Supplementary Figure S2B). We compared the anti-migration activity of three Mortaparibs. As shown in Figure 4A, three Mortaparibs showed comparable anti-migration activity, although they showed different efficacy in proliferation assays as reported earlier [25,26,27,28]. We anticipated that such a high anti-migration activity and low cytotoxicity of Mortaparib^Mild^ could be useful in preventing metastasis in cancer treatment and hence examined its molecular mechanism. As shown in Figure 4B, cells treated with three Mortaparibs showed a decrease in the key regulators of cancer cell migration, including Clic1, hnRNP-k, CARF, vimentin, and mortalin. Of note, Mortaparib^Mild^-treated cells showed a higher reduction in vimentin and mortalin (also confirmed by immunostaining (Figure 4C). Furthermore, a significant decrease in other key regulators of migration and metastasis (including Wnt, CARF, Clic1, hnRNP-k MMP3/10, and β-catenin) was confirmed in cells treated with Mortaparibs (Figure 4C). Similar data were obtained in SKOV3 cells, confirming the high anti-migration activity of Mortaparib^Mild^ (Supplementary Figure S3). Invasion assay in cells treated with three kinds of Mortaparibs showed a significant reduction in treated vs. control cells both in Saos2 and SKOV3 cultures (Figure 5A).

### 3.3. Combinations of Three Mortaparibs Offered Better Anticancer, Anti-Metastasis, and Antistress Activities

**Figure 6:**
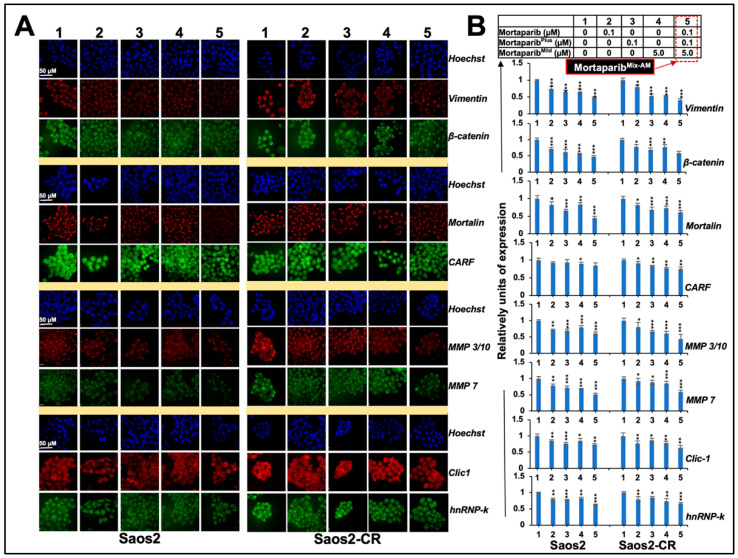
Mortaparib^Mix-AM^ showed strong anti-migration activity. (**A**) Immunostaining of control and treated Saos2/Saos2-CR cells for proteins involved in regulation of cell migration. (**B**) Quantitation of data (mean ± SD) from three independent experiments revealed stronger downregulation of all markers in Mortaparib^Mix-AM^-treated cells. *p* * ≤ 0.05, ** ≤ 0.01, and *** ≤ 0.001 represent significant, very significant, and highly significant, respectively.

Based on previous reports [25,26,27,28] showing that Mortaparib^Mild^ has lower cytotoxicity compared to the other two Mortaparibs, as well as current findings demonstrating its effect on p53-null cells and its significant anti-migration activity at low non-toxic concentrations, we hypothesize that a combination of the three Mortaparibs may provide improved anti-proliferative and anti-migration effects. Such specific combinations may be used to inhibit cancer progression and metastasis, respectively. Various combinations with different ratios of the three Mortaparibs were generated and evaluated for cytotoxicity by MTT assay. At the same time, we also generated cisplatin resistance derivatives of Saos2 cells (Saos2-CR) and examined their response to the Mortaparibs and their mixers. As presented in Supplementary Figure S4A, these derivative cells showed about 20% resistance to cisplatin. However, they responded to the three Mortaparibs almost similarly to the control cells (Figure 5B), also endorsed by the Cyclin D1 and C-Myc immunostaining of control and Mortaparib-treated Saos2 and Saos2-CR cells. As shown in Supplementary Figure S4B, both cells showed equivalent decreases in Cyclin D1 and C-Myc proteins. Based on the MTT-based viability assay of cells treated with either the individual Mortaparibs or their combinations, we identified an anti-proliferative (cytotoxic) combination (#14; called Mortaparib^Mix-AP^ containing 2 μM Mortaparib–0.5 μM Mortaparib^Plus^–10 μM Mortaparib^Mild^) (Figure 5B). The Mortaparib^Mix-AP^ combination showed a synergetic cytotoxic effect as determined by Chou–Talalay’s combination index (CI = 0.81 for Saos2 and 0.74 for Saos2-CR cells). Furthermore, based on our earlier studies and the anti-migration data of Mortaparib^Mild^, as shown in Figure 4, we generated a low-dose non-toxic combination (#5; called Mortaparib^Mix-AM^ containing 0.1 μM Mortaparib–0.1 μM Mortaparib^Plus^–5 μM Mortaparib^Mild^) that did not show any cytotoxicity to either Saos2 or Saos2-CR (Figure 5C) and examined its effect on the cell migration and invasion capability of these cells. As shown in Figure 5D,E, the Mortaparib^Mix-AM^ combination showed a stronger inhibition (as compared to each of the Mortaparibs) of migration and invasion both in Saos2 and Saos2-CR cells; the CI for invasion was 0.29 and 0.34, respectively. Molecular analyses of the markers involved in migration and invasion (vimentin, β-catenin, mortalin, MMP3/10, MMP7, Clic-1, and hnRNP-k) indeed revealed a significantly higher decrease in cells treated with Mortaparib^Mix-AM^ as compared to the ones treated with each of the Mortaparibs individually (Figure 6A,B). Of note, the Saos2-CR cells showed an equivalent response.

**Figure 7:**
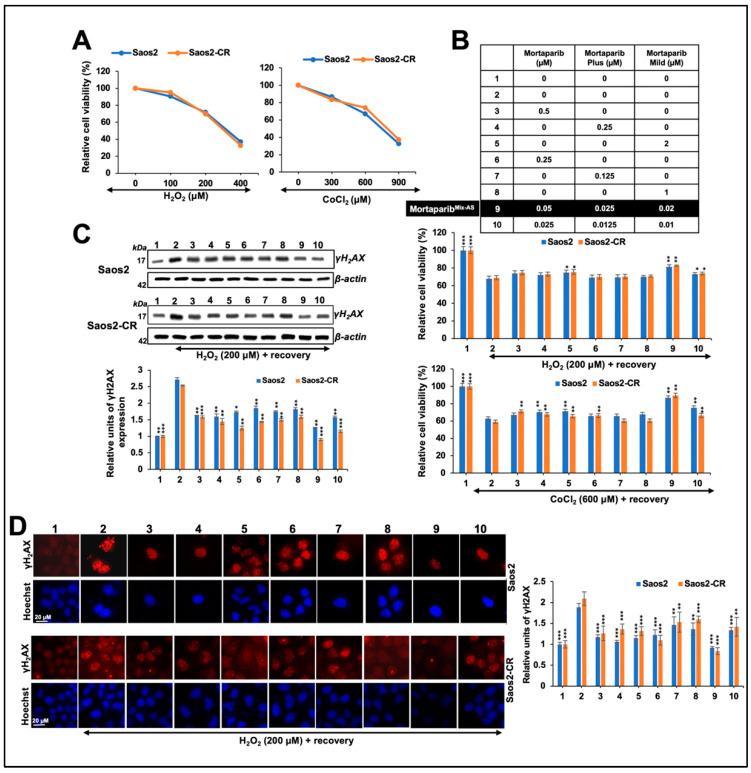
Mortaparib^Mix-AS^ protected cells against oxidative and metal stress. (**A**) Cells were subjected to serially increasing concentrations of H_2_O_2_ for oxidative stress and CoCl_2_ for heavy metal stress. Concentrations that caused 20–30% decrease in viability were selected and treated with Mortaparibs individually (#1–8) or in combination (#9–10). (**B**) Viability of cells subjected to H_2_O_2_ or CoCl_2_, followed by recovery in Mortaparib-supplemented mediums (#3–10), is shown. Cells recovered in #9 (called Mortaparib^Mix-AM^) mixture showed stronger recovery than others. (**C**) DNA damage marker γH2AX in cells subjected to oxidative stress and recovery in either control medium or Mortaparib-supplemented mediums showed strong reduction in presence of Mortaparib^Mix-AM^. The uncropped blots are shown in File S1. (**D**) Immunostaining of γH2AX showing increase in stress cells and recovery in presence of Mortaparib, maximum with Mortaparib^Mix-AS^. Data (mean ± SD) were obtained from three independent biological replicates. *p*-values were calculated using unpaired Student’s *t*-test. * ≤ 0.05, ** ≤ 0.01, and *** ≤ 0.001 represent significant, very significant, and highly significant, respectively.

**Figure 8:**
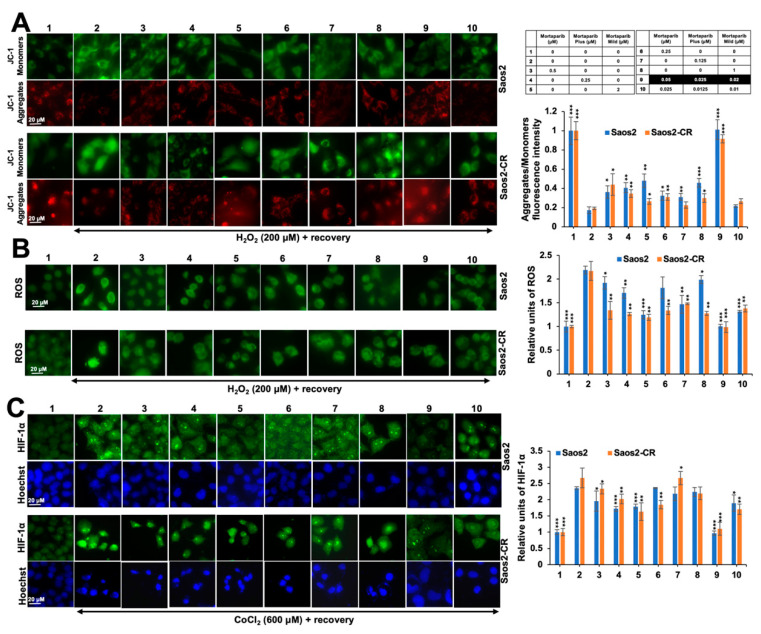
Mortaparib^Mix-AS^ protected cells against mitochondrial dysfunction and ROS accumulation. (**A**) Cells subjected to oxidative stress and recovered in medium supplemented with Mortaparibs (#3–10) were examined for mitochondrial membrane potential. Stressed cells recovered in control medium showed loss of JC-1 aggregates (#2) and their recovery in presence of Mortaparibs, maximum in #9 (Mortaparib^Mix-AS^). (**B**) ROS staining in control, stressed, and recovered cells showing maximum recovery in #9 (Mortaparib^Mix-AS^) group. (**C**) Immunostaining of control, stressed, and recovered cells for HIF-1α showing its increase in cells exposed to CoCl_2_ and recovery in medium supplemented with Mortaparibs, maximum with Mortaparib^Mix-AS^. Data (mean ± SD) were obtained from three independent biological replicates. *p*-values were calculated using unpaired Student’s *t*-test. * ≤ 0.05, ** ≤ 0.01, and *** ≤ 0.001 represent significant, very significant, and highly significant, respectively.

Based on our earlier findings that low doses of anticancer compounds have antistress activity [29], we next determined if the combination of Mortaparibs could provide antioxidative and anti-hypoxia stress activities. Initially, cells were challenged with H_2_O_2_ or CoCl_2_ to a level that caused ~ 30% reduction in viability upon recovery in the complete medium (Figure 7A). Based on these assays, 200 μM H_2_O_2_ and 600 μM CoCl_2_ were selected. Stressed cells were recovered in eight kinds of Mortaparib-supplemented medium. As shown in Figure 7, we identified a combination (#9, called Mortaparib^Mix-AS^ containing 0.05 μM Mortaparib–0.025 μM Mortaparib^Plus^–0.02 μM Mortaparib^Mild^) that caused a better recovery of cells challenged with either oxidative or heavy metal stress (Figure 7B). The combination showed a synergetic effect in both cell and stress types; the CI was <0.25 in each case. Molecular analyses of whole cell lysates of control and oxidatively stressed cells revealed an increase in γH2AX in the latter (Figure 7C). Stressed cells recovered in a medium supplemented with low non-toxic concentrations of Mortaparibs showed the attenuation of γH2AX increase. Of note, Mortaparib^Mix-AS^-treated cells showed a strong reduction and normalization of γH2AX (Figure 7C) and were supported by the immunostaining of γH2AX foci (Figure 7D). Similar protection against mitochondrial dysfunction and ROS accumulation was detected. Whereas oxidatively stressed cells showed the depolarization of the mitochondrial membrane (as determined by decreased red-JC1 staining) (Figure 8A) and accumulation of ROS (Figure 8B), cells recovered in the Mortaparib-supplemented medium showed recovery in both parameters. Of note, Mortaparib^Mix-AS^-treated cells showed the normalization of the mitochondrial membrane potential and ROS. Furthermore, the response of the Soas2-CR cells was similar to that of Saos2 (Figure 8A,B). We determined the HIF-1α expression level in cells exposed to CoCl_2_. As shown in Figure 8C, the CoCl_2_-induced increase in HIF-1α was attenuated in cells treated with Mortaparibs, leading to complete recovery in cells treated with Mortaparib^Mix-AS^.

## 4. Discussion

The role of mortalin enrichment in carcinogenesis, malignant transformation, drug resistance, and cancer cell stemness has been supported by several independent studies on a variety of cancer models [11,14,21,24,30,31,32,33,34,35]. In consensus, mortalin-compromised cancer cells undergo growth arrest or apoptosis involving diverse mechanisms. For example, the reactivation of the tumor suppressor activity of p53 by the disruption of its interaction with mortalin [11,36], aberrant transport and misfolding of polypeptides [37], ATP depletion, and mitochondrial dysfunction leads to intrinsic apoptosis [11,14,38,39]. Earlier studies have also shown that the abrogation of mortalin–p53 interaction in cancer cells with wild-type p53 triggers growth arrest, and the cells with mutant p53 undergo apoptosis [11,38,39,40] suggesting mortalin–p53 interaction can be an effective therapeutic target in cancer cells irrespective of their p53 status.

In light of the above reports, mortalin inhibitors are anticipated as candidate anticancer drugs. Earlier, MKT-077 (a cationic rhodacyanine dye analog) and its derivatives were shown to cause selective toxicity to cancer cells by binding to mortalin and disrupting its interactions with p53 in various models [11,41,42]. Other cellular targets of MKT-077 include oncogenic Ras, F-actin, and HIF-1α [11,39]. Mortalin depletion was shown to cause the selective death of tumor cells with K-RasG12V mutation by the mitochondria-mediated death pathway independent of the activation of p53 and p21 [23,33]. Similar effects were obtained with the MKT-077 derivative (JG-98) in in vitro and in vivo mice models suggesting mortalin to be a selective target for oncogenic KRAS tumors [43]. Most recently, MKT-077 analogs (JG-98 and JG-194) that show higher selectivity to mortalin and better bioavailability were tested for their effect on medullary thyroid carcinoma (MTC) cell lines using three-dimensional culture assays. The compounds not only suppressed the growth of MTC cell lines but also their derivatives that were resistant to vandetanib and cabozantinib. The findings emphasized the inhibition of mortalin as an effective therapeutic strategy for drug-resistant cancer cells [43]. In line with this, cancer cell stemness markers (ABCG2, OCT-4, CD133, ALDH1, CD9, MRP1, and connexin) that contribute to drug resistance were found to correlate with mortalin overexpression in cancer cells, and anti-mortalin small molecules, including shRNA, MKT-077, SHetA2 and CAPE, sensitized these cells to chemotherapeutic drugs [11,38].

In agreement with the essential role of mortalin in cancer metastasis, its inhibition has been shown to compromise the migration, invasion, and angiogenic characteristics of metastatic cancer cells through the inactivation of EMT signaling [11,18,20,24,34]. Similar to these studies, cancer cells treated with noncytotoxic doses of Mortaparibs were compromised in their metastatic characteristics and also involved p53-independent pathways (Figure 4, Figure 5 and Figure 6) [25,26,27]. Most recently, mortalin was seen to bind directly to HIF-1α (Hypoxia-inducible factor-1, an essential factor of activated hypoxia signaling in metastatic tumors) and block the Bax-mediated apoptosis of cancer cells [39]. In silico studies on the binding domains of mortalin, p53, and small molecule inhibitors have revealed the potential of several natural compounds (Withaferin A, Withanone, Cucurbitacin, Fucoxanthin, and CAPE) to serve as mortalin inhibitors [11,18,19,20] awaiting their validation in clinical studies.

The clinic success of anticancer drugs is marked by inhibitors of PARP1 (Poly ADP-ribose polymerase 1, a major sensor and central player of DNA damage response and repair mechanisms, DDR) that play a vital role in maintaining the genomic stability of normal cells. Defective DDR and the accumulation of DNA damage are established hallmarks of tumorigenesis and its progression to aggressive stages. Several FDA-approved PARP1 inhibitors (Olaparib, Niraparib, Rucaparib, Talazoparib, Fuzuloparib, and Pamiparib), although limited by the development of resistance, are used for treating a wide range of cancers [44,45,46,47,48]. Molecular docking analyses have shown earlier that Mortaparib^Mild^ has the capability of interacting directly with the catalytic binding domain of PARP1 [28]. Furthermore, mortalin and PARP1 have been shown to interact by molecular docking as well as co-immunoprecipitation assays [25]. Mortalin overexpression and knockdown have been shown to cause an increase and decrease in PARP1 activity, respectively, and suggested a tight association of the two proteins [25]. In these perspectives, Mortaparibs that target mortalin and PARP1 simultaneously [25,26,27,28] are anticipated to offer clinical advantages. Indeed, the binding of Mortaparib to PARP1 has been compared with its three known inhibitors, Olaparib, Rucaparib, and Niraparib, in an earlier study that reported similar binding sites, orientations, and the affinities of the four compounds [25] suggesting the potential of Mortaparib as a PARP1 inhibitor. As shown in Figure 2 and Figure 3 [25,26,27,28], Mortaparib^Mild^-treated cells showed the downregulation of mortalin and PARP1, compromised PARP1 function, and decrease in PAR and its trapping into DNA in p53-null cells suggesting its application in cancer irrespective of the p53 status. Such a p53-independent role of mortalin in several key pathways of carcinogenesis has also been endorsed in earlier studies [11,14,20,49,50]. We anticipated that the combination of the three Mortaparibs may be able to recruit more cancer targets, as also suggested by earlier studies [26,27], and thus generate their combination (Mortaparib^Mix^). Considering the positive correlation of chronic stress with cancer, metastasis, and relapse [5,6,51], we further formulated combinations of the three Mortaparibs (Mortaparib^Mix^) for their specific use targeting either the proliferation, migration, or stress phenotype of cancer cells. As shown in Figure 5, Figure 6, Figure 7 and Figure 8, while Mortaparib^Mix-AP^ showed higher cytotoxicity, Mortaparib^Mix-AM^ and Mortaparib^Mix-AS^ showed better anti-metastasis and antistress effects, respectively. Of note, as demonstrated by phenotypic and extensive molecular marker analyses, drug-resistant derivative cells were also seen to respond to the combinations, suggesting their potential in treating cancer and its subsequent recurrence. Collectively, we report that a combination of Mortaparibs may offer a new strategy to target multiple functions of mortalin and PARP1 involved in carcinogenesis, its progression, and relapse.

## 5. Conclusions

The small molecule inhibitor of mortalin, Mortaparib^Mild^, blocked the proliferation, migration, and stress characteristics of p53-null cancer cells through the downregulation of mortalin, PARP1, and several other key regulators of these phenotypes. Combinations (Mortaparib^Mix-AP^, Mortaparib^Mix-AM^, and Mortaparib^Mix-AS^) of the three Mortaparibs offered high potency in targeting the proliferation, migration, and stress characteristics of cancer cells. This study proposes (i) the wider use of Mortaparibs in the treatment of cancers with different genetic backgrounds and (ii) their mixture (Mortaparib^Mix^) customized to target proliferation, migration, or stress characteristics in cancer treatment modalities.

## Data Availability

All datasets used and/or analyzed during the current study are available in the manuscript and Supplementary Materials.

## Supplementary Materials

The following supporting information can be downloaded at: https://www.mdpi.com/article/10.3390/cancers16122239/s1, Figure S1: Immunostaining, cell cycle, and flow cytometric analyses of control and treated cells; Figure S2: Images showing cell morphology and wound-scratch assay of both control and treated cells; Figure S3: Wound-scratch assay and immunostaining of control and treated cells for proteins involved in migration; Figure S4: Viability and immunostaining of control and treated cells; Table S1. List of primary antibodies (and Refs. [52,53]) used for Western blotting and immunostaining; Table S2. Composition of the trapping assay buffers; File S1: Figures S5–S9, Full pictures of the Western blots.
