# Supplementary material for: Mixtures of Three Mortaparibs with Enhanced Anticancer, Anti-Migration, and Antistress Activities: Molecular Characterization in p53-Null Cancer Cells
Source: Cancers (Basel). 2024 Jun 17;16(12):2239. doi: 10.3390/cancers16122239 (PMC11202144; doi:10.3390/cancers16122239)

Mixtures of Three Mortaparibs with Enhanced Anticancer, Antimigration, and Antistress Activities: Molecular Characterization in p53 null Cancer Cells

Renu Wadhwa[$*], Shi Yang[$], Hazna Noor Meidinna, Anissa Nofita Sari[1], Priyanshu Bhargava[2], Sunil C Kaul[*]

AIST-INDIA DAILAB, National Institute of Advanced Industrial Science & Technology (AIST), Central 4-1, Tsukuba 305-8565, Japan
[$]Authors contributed equally to this work
Current affiliation - [1]Research Center for Vaccine and Drugs, National Research and Innovation Agency (BRIN), Jalan Raya Jakarta-Bogor KM46, Cibinong, Bogor 16911, Indonesia and [2]Division of Life Science, The Hong Kong University of Science and Technology, Clear Water Bay, Kowloon, Hong Kong, SAR, China
*Correspondence: renu-wadhwa@aist.go.jp (R.W.); s-kaul@aist.go.jp (S.C.K.)

Full uncropped Western blots presented in Figures 2A, 3A, 3B, 3D, 4B, and 7C

Figure S5. Full uncropped Western blots for the protein of interest (Mortalin) in control and Mortaparib^{Mild}-treated Saos2 and SKOV3 cells (**Figure 2A**). β-actin was used as an internal loading control.

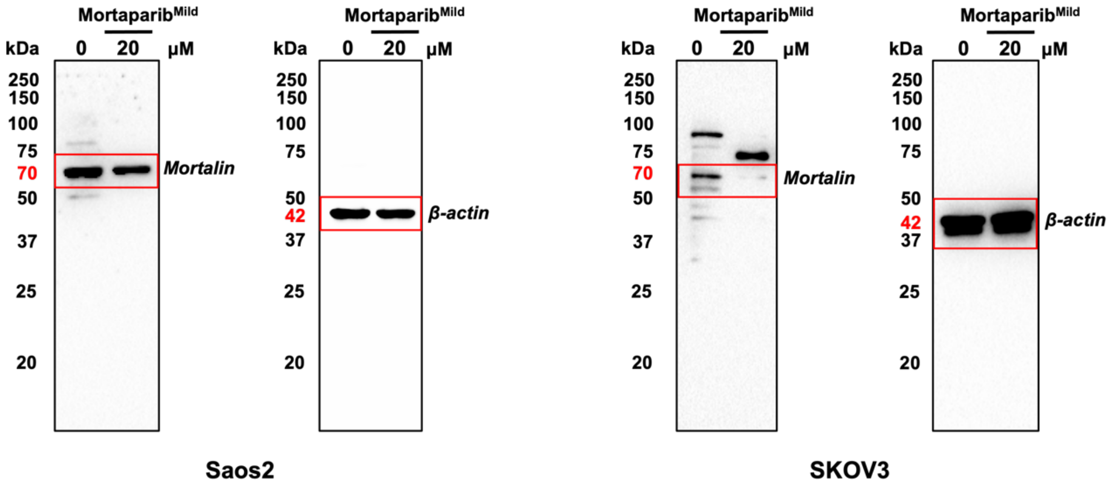

Figure S6. Full uncropped Western blots for the protein of interest (PARP-1, Cleaved PARP-1, PAR) were detected in control and Mortaparib^{Mild}-treated Saos2 and SKOV 3 cells (**Figures 3A and 3B**). β-actin was used as an internal loading control.

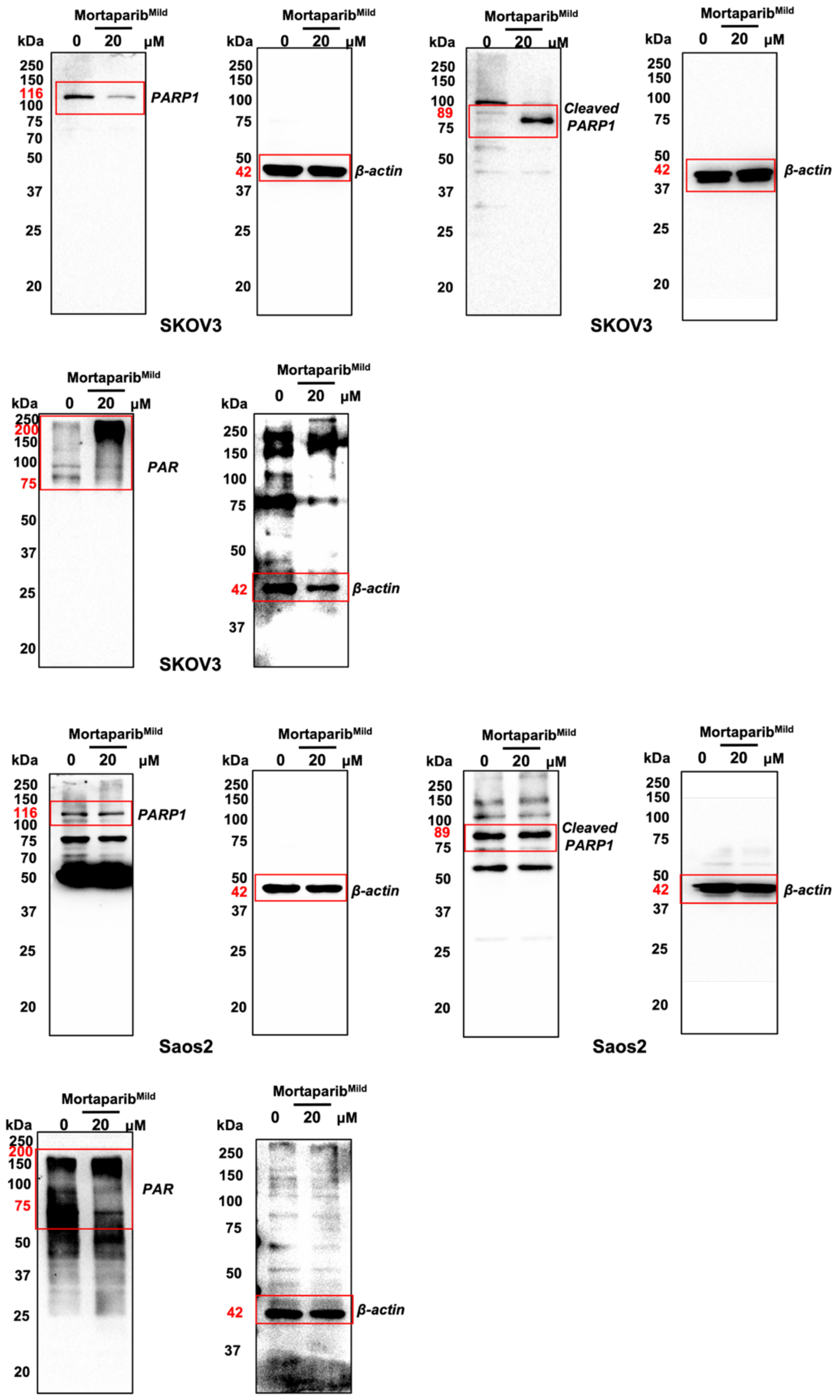

Figure S7. Full uncropped Trapping Assay for the protein of interest (PARP-1) detected from control and Mortaparib^{Mild}-treated Saos2 cell lysates (**Figure 3C**). H3 was used as an internal loading control.

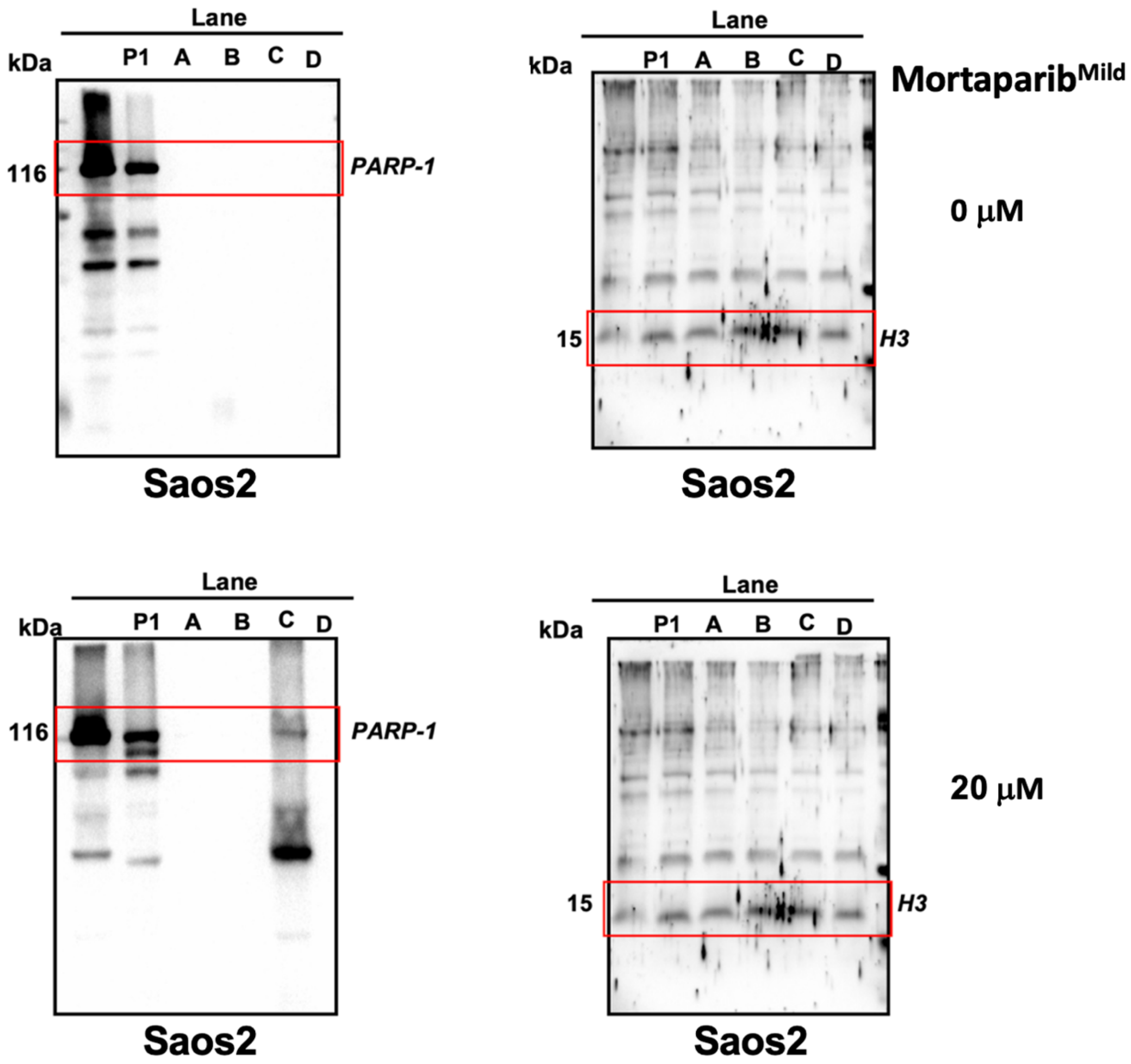

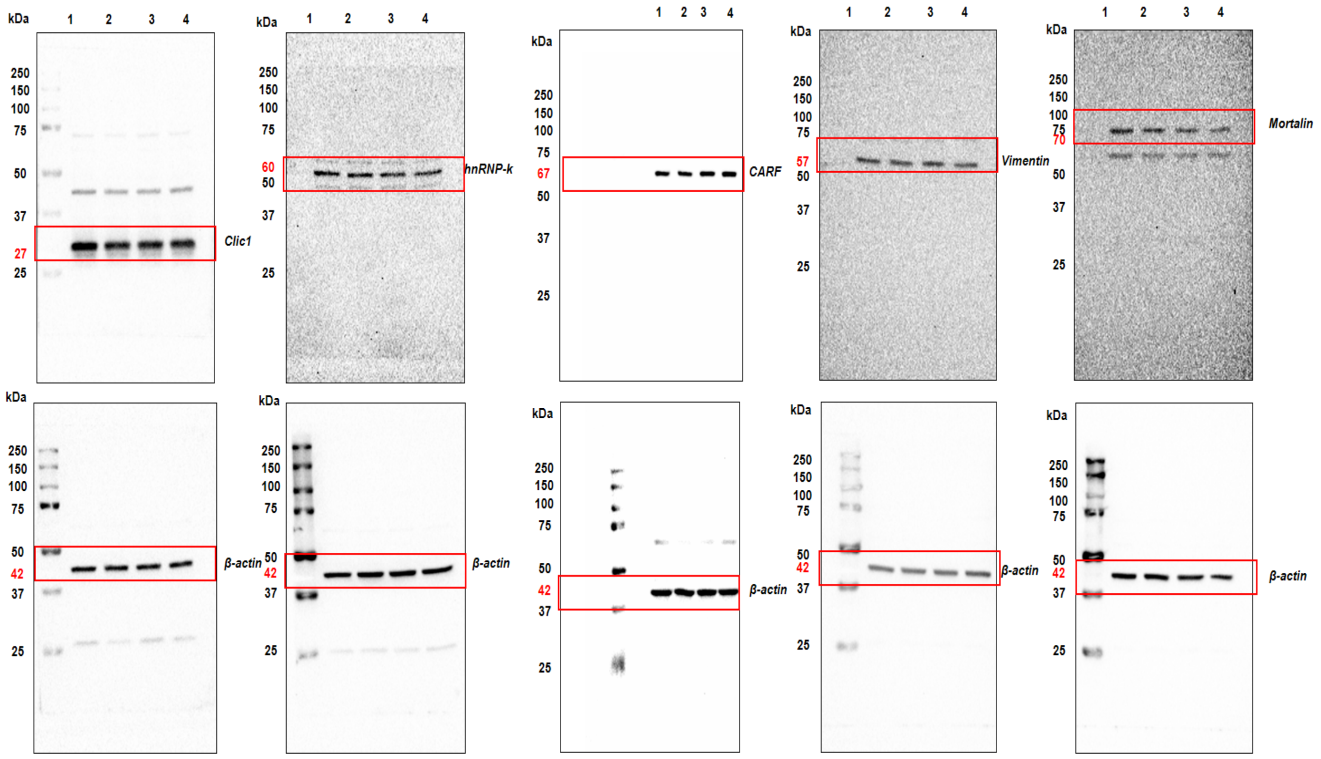

Figure S8. Full uncropped Western blots for the proteins of interest (Clic1, hnRNP-k, CARF, Vimentin, and Mortalin) detected from Mortaparib class-treated and control Saso2 cell lysates (**Figure 4B**). β-actin was used as an internal loading control.

Figure S9. Full uncropped Western blots for the proteins of interest (γH2AX) were detected from control and Mortaparib-treated Saso2 and Saos2-CR cells (**Figure 7C**). β-actin was used as an internal loading control.

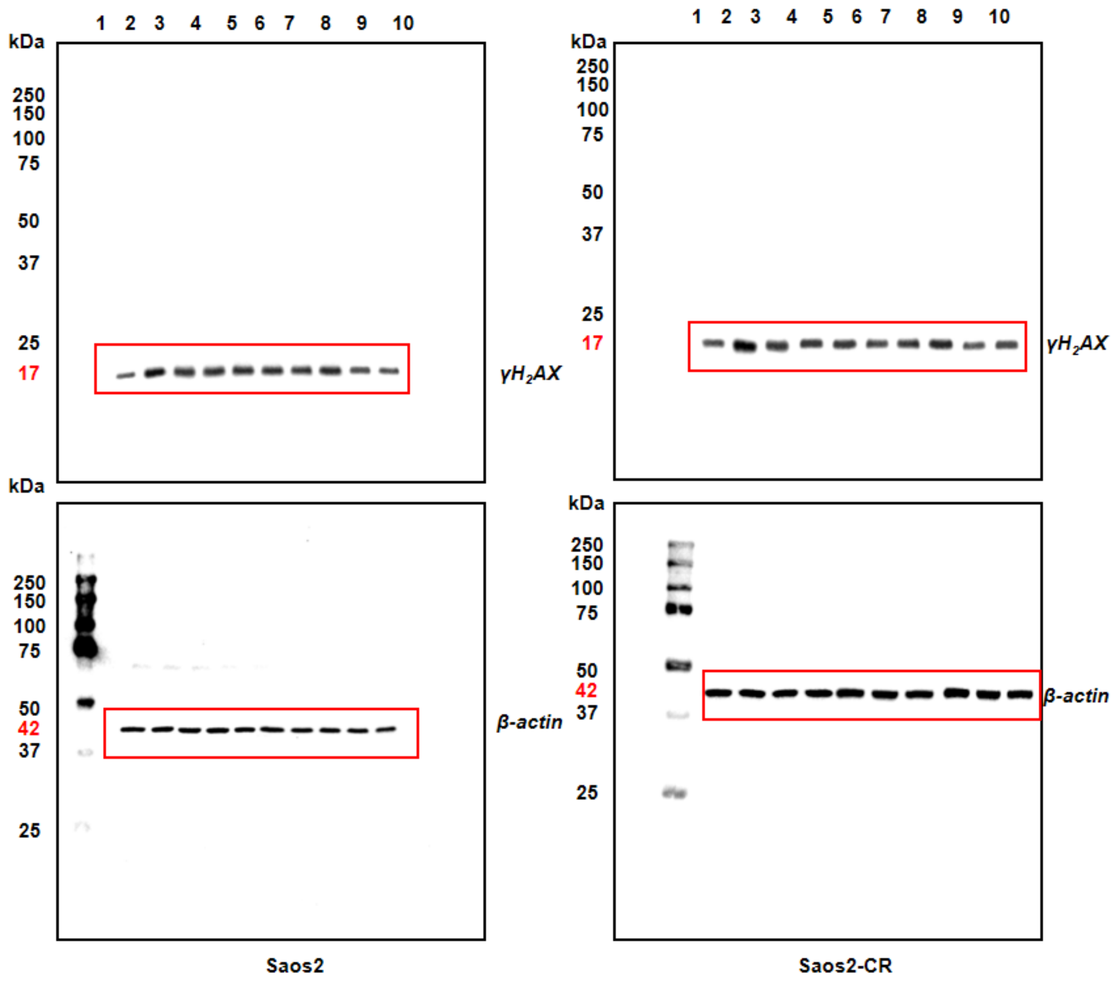

Supplement: Supplementary file 1 [file cancers-16-02239-s001.zip › cancers-3032094-File S1. Original Images for Blots.pdf]
