# Supplementary material for: Mixtures of Three Mortaparibs with Enhanced Anticancer, Anti-Migration, and Antistress Activities: Molecular Characterization in p53-Null Cancer Cells
Source: Cancers (Basel). 2024 Jun 17;16(12):2239. doi: 10.3390/cancers16122239 (PMC11202144; doi:10.3390/cancers16122239)

SUPPLEMENTARY INFORMATION

Mixtures of Three Mortaparibs with Enhanced Anticancer, Anti-migration, and Antistress Activities: Molecular Characterization in p53-Null Cancer Cells

Renu Wadhwa [*,†], **Shi Yang** [†], **Hazna Noor Meidinna**, **Anissa Nofita Sari** [‡], **Priyanshu Bhargava** [§] **and Sunil C. Kaul** [*]

AIST-INDIA DAILAB, National Institute of Advanced Industrial Science & Technology (AIST), Central 4-1, Tsukuba 305-8565, Japan; s2036036@u.tsukuba.ac.jp (S.Y.); hazna.sec@alirsyadsatya.sch.id (H.N.M.); anissa.nofita.sari@brin.go.id (A.N.S.); bhargava@ust.hk (P.B.)

* Correspondence: renu-wadhwa@aist.go.jp (R.W.); s-kaul@aist.go.jp (S.C.K.)

† These authors contributed equally to this work.

‡ Current address: Research Center for Vaccine and Drugs, National Research and Innovation Agency (BRIN), Jalan Raya Jakarta-Bogor KM46, Cibinong, Bogor 16911, Indonesia.

§ Current address: Division of Life Science, The Hong Kong University of Science and Technology, Clear Water Bay, Kowloon, Hong Kong SAR, China.

Additional files

Table S1. List of primary antibodies used for Western blotting and immunostaining.

Antibody	Source	Catalog No.	Antibody	Source	Catalog No.
Wnt-1	Santa Cruz	sc-514531	c-Myc	Abcam	ab32072
MMP-3/10	Santa Cruz	sc-374029	Clic1	Santa Cruz	sc-81873
γH2AX	Cell Signaling	9718s	HIF-1α	Novus Biologicals	NB100-479
β-catenin	Santa Cruz	sc-7963	Cleaved PARP1	Cell Signaling	9541s
hnRNP-k	Cell Signaling	4675	p53	Santa Cruz	sc-126
E-cadherin	Cell Signaling	14472	PARP1	Santa Cruz	Sc-7150
N-cadherin	Cell Signaling	13116	p21	Cell Signaling	2947s
Vimentin	Santa Cruz	sc-6260	Cdk4	Santa Cruz	sc-260
CARF	Raised in our lab [1]	A-10	Cyclin D1	Santa Cruz	sc-56302
Mortalin	Raised in our lab [2]	37-6			

1. Kalra, R.S.; Chaudhary, A.; Omar, A.; Li, X.; Khurana, M.; Kaul, S.C.; Wadhwa, R. Stress-induced changes in CARF expression serve as a quantitative predictive measure of cell proliferation fate. *Exp Cell Res* **2023**, *429*, 113669, doi:10.1016/j.yexcr.2023.113669.
2. Shiota, M.; Ikeda, Y.; Kaul, Z.; Itadani, J.; Kaul, S.C.; Wadhwa, R. Internalizing antibody-based targeted gene delivery for human cancer cells. *Hum Gene Ther* **2007**, *18*, 1153-1160, doi:10.1089/hum.2007.087.

Table S2. Composition of the trapping assay buffers.

Buffer	Ingredients
Hypotonic	100 mM MES-NaOH pH 6.4, 1 mM EDTA, 0.5 mM $MgCl_2$, 30% sucrose in Mili-Q H_2O
Buffer A	50 mM HEPES-NaOH pH 7.5, 100 mM KCl, 2.5 mM $MgCl_2$, 0.05% Triton X-100
Buffer B	50 mM HEPES-NaOH pH 7.5, 250 mM KCl, 2.5 mM $MgCl_2$, 0.05% Triton X-100
Buffer C	50 mM HEPES-NaOH pH 7.5, 500 mM KCl, 2.5 mM $MgCl_2$, 0.1% Triton X-100
Buffer D	Buffer A, 5 mM CaCl2, Micrococcal protease inhibitor three-unit (Roche Diagnostic GmbH, Mannheim, Germany)

Supplementary Figure S1 - (A) Immunostaining of control and Mortaparib^{Mild} treated p53 null cancer cells (Saos2 and SKOV3) for p53 (red). U2OS (p53 wild type) was used as a control. Cells were stained with Hoechst 33342 for nuclear staining (blue). Quantitation of the staining expressed as relative units of p53 is shown on the right. (**B)** Cell cycle analysis of control and Mortaparib^{Mild} treated cells showed an increase in cell population at G2 arrest and was more pronounced in SKOV3 as compared to Saos2. (**C)** Flow cytometric analysis showing a dose-dependent increase in early apoptotic cells in Mortaparib^{Mild} treated both Saos2 and SKOV3 cultures.

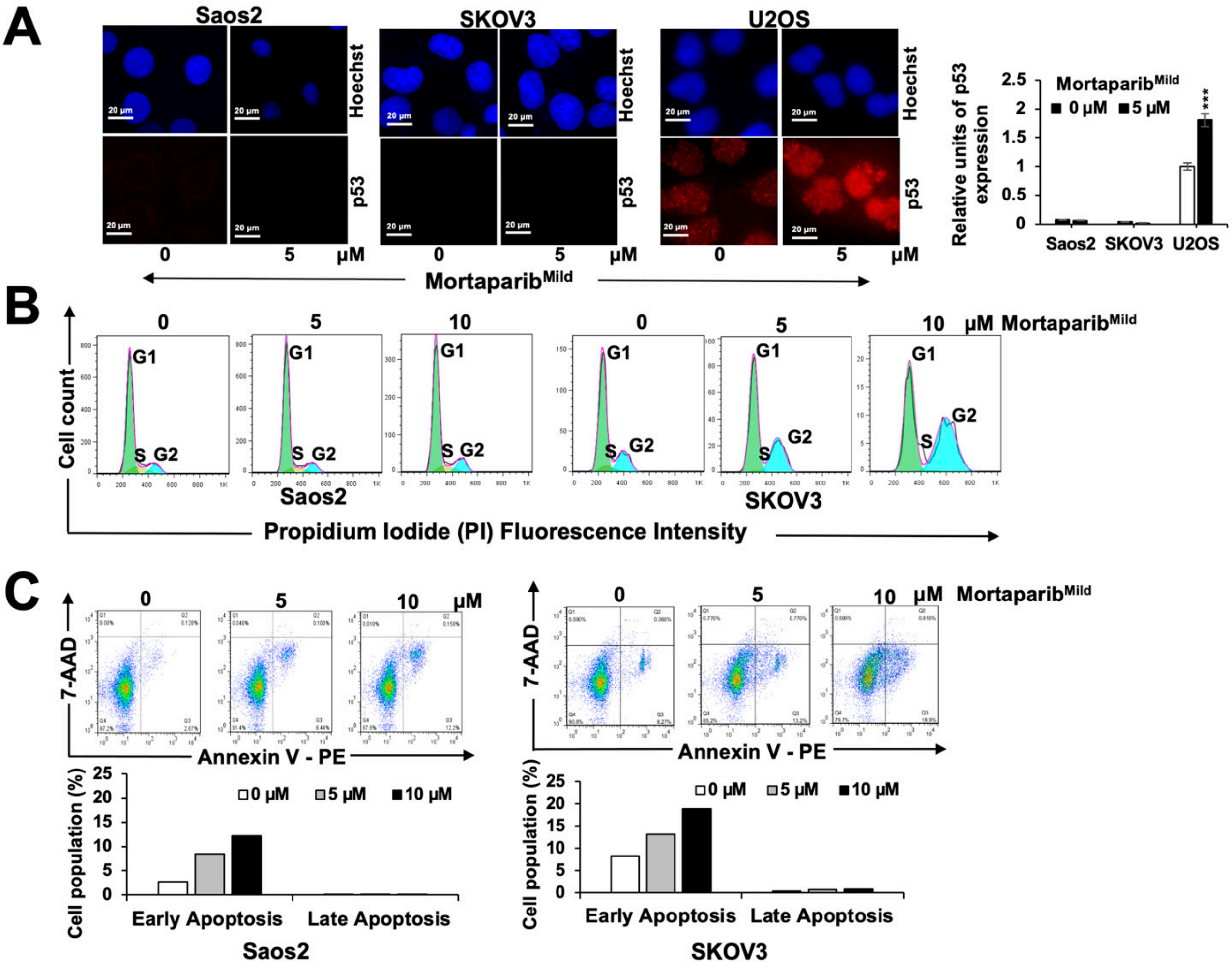

Supplementary Figure S2 - (A) Phase contrast pictures showing the morphology of control and Mortaparib^{Mild} treated Saos2 and SKOV3 cells. Apoptotic-like morphology was seen in cells treated with 10 and 20 mM. Cultures treated with 5mM did not show such an effect. (**B)** Wound-scratch assay of control and Mortaparib^{Mild} (5mM) treated cells showed a delay in migration of the treated cells into the wound. Quantitation of the data from three independent experiments is shown; p-values were calculated using an unpaired Student's t-test. * ≤0.05, ** ≤0.01, and ***≤0.001 represent significant, very significant, and highly significant, respectively.

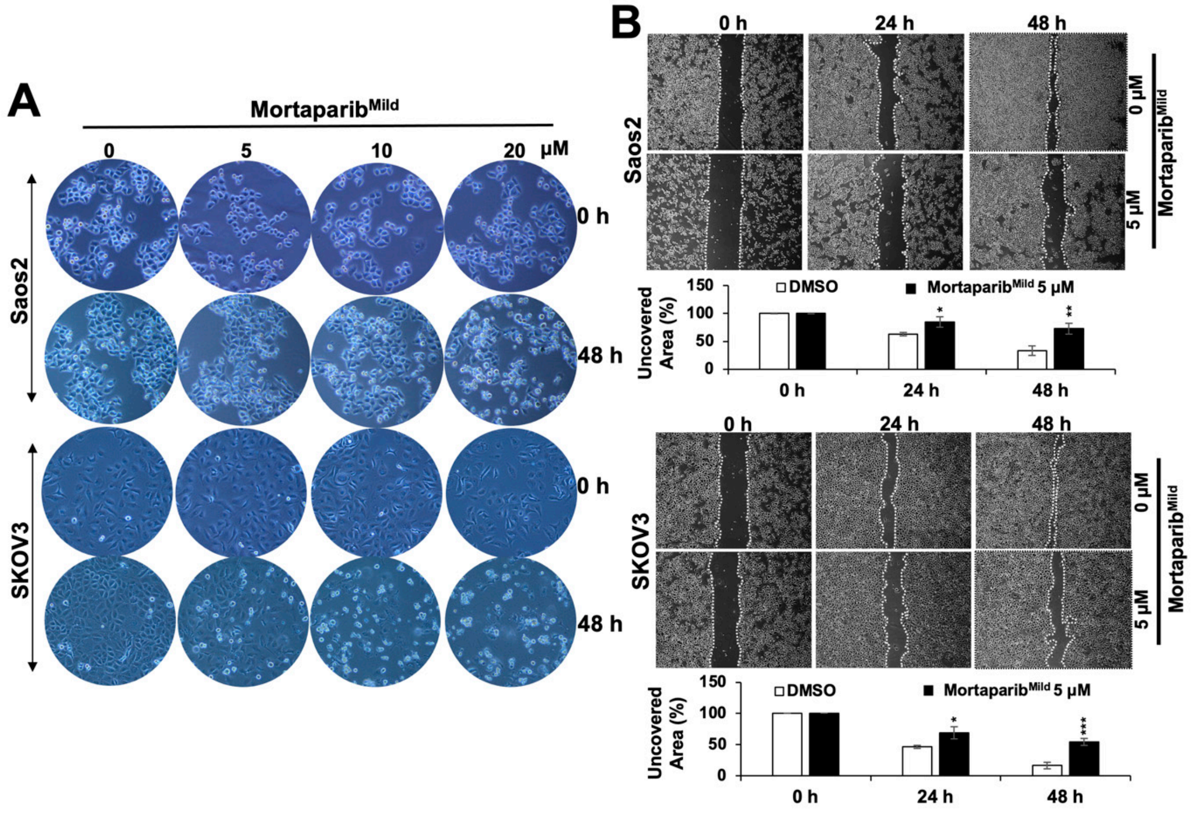

Supplementary Figure S3 - (A) Wound-scratch assay in SKOV3 cells treated with nontoxic doses of mortaparibs is shown. The treated groups showed a delay in migration. Quantitation of the data is shown below. **(B)** Immunostaining of control and treated cells for proteins involved in migration. Quantification is shown left (Below). Data (mean ± SD) was obtained from three independent experiments. The p-values * ≤0.05, ** ≤0.01, and ***≤0.001 represent significant, very significant, and highly significant, respectively.

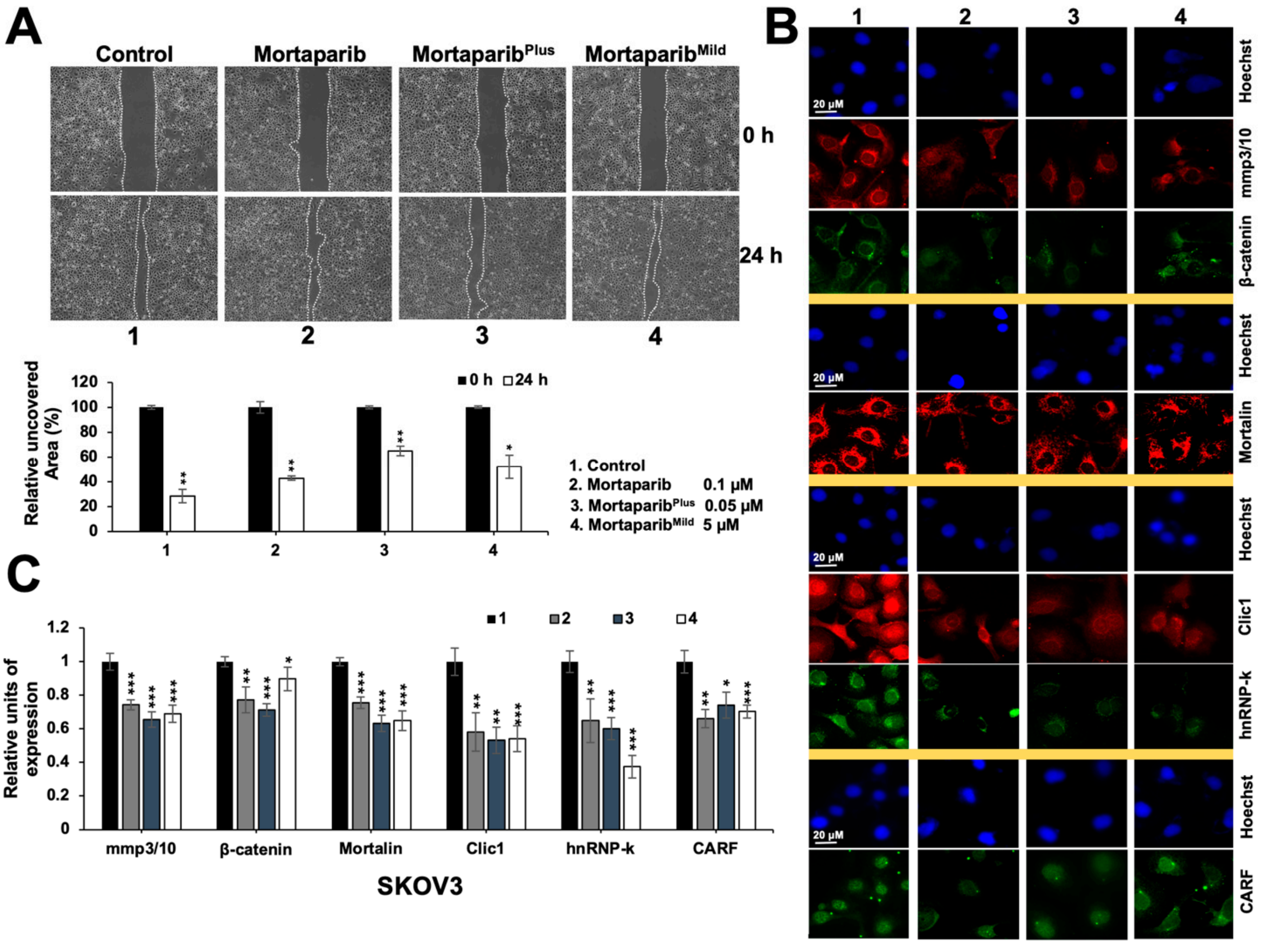

Supplementary Figure S4 - (A) Viability of cells cultured in control or cisplatin-supplemented DMEM for about 2 months. **(**B)Immunostaining of Saos2 and Saos2-CR treated with three mortaparibs for Cyclin-D1 and C-myc showed comparable responses of the two cell lines.

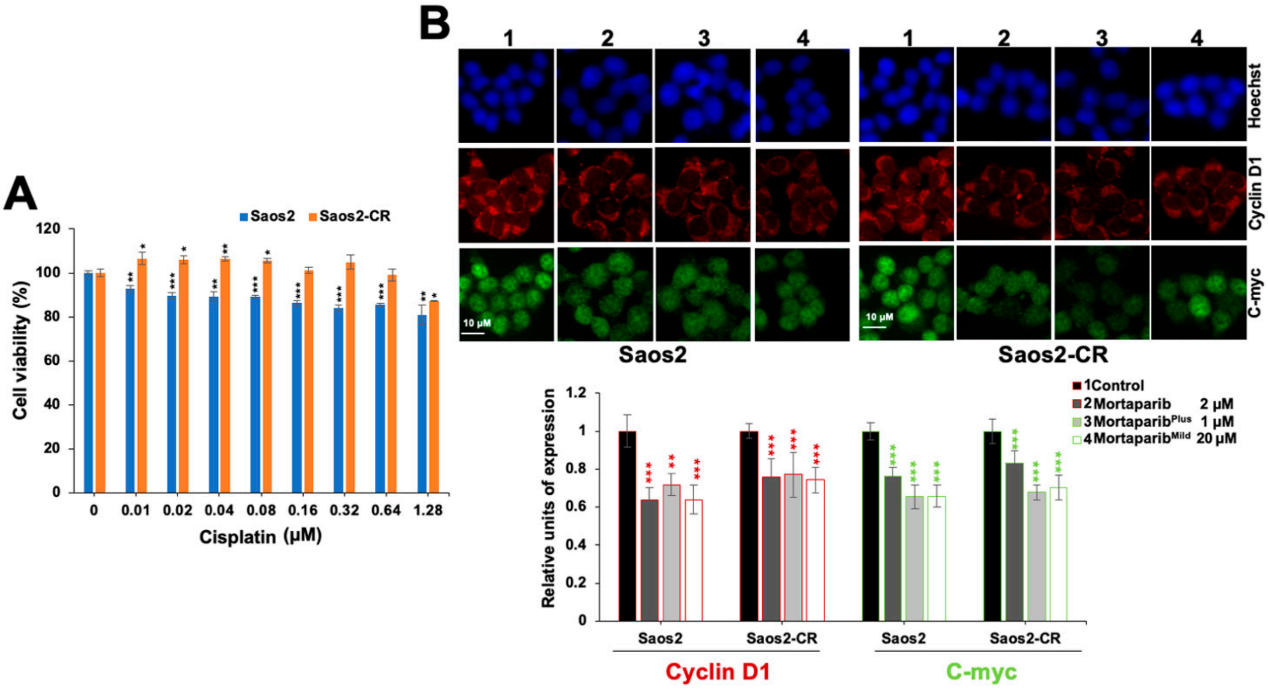

Supplement: Supplementary file 1 [file cancers-16-02239-s001.zip › cancers-3032094-supplementary.pdf]
